# Use of Rapid Capillary Zone Electrophoresis to Determine Amino Acids Indicators of Herring Ripening during Salting

**DOI:** 10.3390/foods10112518

**Published:** 2021-10-20

**Authors:** Katarzyna Felisiak, Mariusz Szymczak

**Affiliations:** 1 Department of Fish, Plant and Gastronomy Technology, Faculty of Food Sciences and Fisheries, West Pomeranian University of Technology, 71-459 Szczecin, Poland; kfelisiak@zut.edu.pl; 2 Department of Toxicology, Dairy Technology and Food Storage, Faculty of Food Sciences and Fisheries, West Pomeranian University of Technology, 71-459 Szczecin, Poland

**Keywords:** capillary electrophoresis, salting, herring, amino acids, ripening, trichloroacetic acid extracts

## Abstract

Currently, herring fillets are salted with acetic acid to activate muscle proteases. This causes a change in the composition of free amino acids, compared to salting of whole fish with viscera proteases. Therefore, old indicators of the ripening dynamics of salted fish based on amino acids are not current. Determination of free amino acids can be performed by many methods, but most are labor intensive and expensive. Therefore, a capillary electrophoresis method without derivatization (CZE) was used to determine the actual ripening rates of salted herring fillets. A group of hydrophobic and basic amino acids were determined in trichloroacetic acid (TCA) extracts of meat and brine to develop 16 indicators. Statistical regression analysis of the indicators (R2adj, RMSE, cluster analysis) followed by principal component analysis (PCA) correlation analysis of the indicators vs sensory evaluation parameters of texture and TPA-hardness of salted fillet meat allowed the choice of the most precise indicators. The best indicator in meat was Phe/Tyr-height, which value increased during salting. A more precise indicator of ripening was His/Tyr-height in brine, which value decreased during salting. Sensory evaluation parameters of salted herring texture correlated strongly with TPA-hardness and traditional indicators such as non-protein nitrogen and protein hydrolysis product fraction content. However, the most precise indicators were those obtained from amino acids determined by the CZE method. Results obtained in this study may be suitable for fast monitoring of the salted herring ripening process in industry.

## 1. Introduction

Salting is one of the popular methods of clupeid fish preserving. The ready-to-eat product obtains the desired sensory characteristics and high nutritional value including easily digestible protein and polyunsaturated fatty acids [1,2]. During salting, changes in salt content and protein hydrolysis products (PHP) occur in fish. The proportion of free amino acids (FAA) and peptides in the meat of salted fish is characteristic. Amino acids have their own characteristic taste, which is why they are responsible for the specific taste of ripened salted fish meat [3,4,5]. Quantitative and qualitative composition of PHP determines the sensory quality, nutritive value, and a high content of biologically-active compounds of a ripened fish product [1,6]. During protein breakdown the texture of meat is also changed. The ripening rate of brine-salted fish depends mainly on the fishing season, endogenous enzyme activity, salt concentration and salting process temperature [7,8,9]. To determine the ripeness rate of salted herring, sensory analysis is used as well as the quantitative composition of PHP by chemical methods, which are labor- and time-consuming [10]. Kiesvaara [3] and Mendes et al. [5] have proposed for this purpose designation of FAA-basic to FAA-acidic ratio. Many instrumental methods allow the determination of amino acids, but require special sample preparation and expensive analyses, making these methods not routinely used in industry.

The ripening rates to date have been determined for traditional whole fish salting methods in which the meat ripens primarily due to the activity of digestive proteases [11,12,13]. Nowadays in the industry, herring fillets are usually salted instead of whole herring. Therefore, currently, ripening of salted herring fillets occurs mainly due to the activity of muscle proteases called cathepsins [7,14]. To stimulate acidic muscle proteases, an approximately 1% addition of acetic acid is used [15,16]. At the same time, lowering the pH value of fillet meat inhibits the activity of possibly diffusible digestive enzymes into the meat, such as trypsin, chymotrypsin, and basic aminopeptidases. Trypsin prefers to cleavage the positively charged amino acid side chain, while chymotrypsin prefers aromatic side chains. Fish trypsin has shown that elevated levels of amino acids such as glutamate, alanine, leucine, serine, lysine, arginine, and proline add positive flavor to the seafood product [17]. Chymotrypsin prefers release of valine, leucine, isoleucine, methionine, phenylalanine and tyrosine, but relative amounts of single free amino acids stay unchanged [7]. In turn, muscle cathepsin activity promotes the release of aspartic acid, threonine, proline, glycine, tyrosine, lysine, and serine [18]. Differences in herring ripening between the traditional and the present method are due to the influence of table salt, which activates digestive proteases and inhibits the activity of cathepsins B, E, D, except for cathepsin L [19]. The use of a different group of proteases leads to a different amino acid composition in salted herring and a change in the taste of these products. Kołakowski and Bednarczyk [15,16] and Szymczak and Kołakowski [20] showed that using a low concentration of acetic acid promotes the loss of PHP (mainly amino acids) from the meat to the surrounding brine. These losses are greater at low salt concentration in the muscle (<6%) where the solubility of muscle proteins is relatively high [21]. The compounds in brine are a “mirror image” of the processes occurring in the flesh of salted fish, with some differences. The advantages of the brine test compared to meat are greater repeatability of results and no need to damage the product [19,22,23]. Therefore, the existing ripening indicators of salted fish meat based on free amino acids need to be revised.

Finding an objective method for monitoring the salted herring ripening process is still a challenge. A method that allows rapid profiling of protein hydrolysis products is capillary zone electrophoresis (CZE) [24,25,26,27]. Although high salt concentrations in the sample are problematic in CZE analysis, a novel CZE method has been developed that enables correct separation of PHPs from TCA extracts of salted fish [28,29]. Therefore, the aim of this study was to determine the best indicators of meat ripening of herring fillets salted with the current acid-method, based on the content of selected amino acids in TCA extracts of meat and brine, using the CZE method.

## 2. Materials and Methods

### 2.1. Salted Herring

Whole herring used were 236.5 ± 7.7 cm and 157.6 ± 12.0 g, and fillets 78 ± 8.4 cm. Herring before salting had a freshness grade of 5.10 ± 0.4 points as determined by the QIM method [30]. Fresh herring was gutted, washed and filleted manually in laboratory. Fillets of Baltic herring (*Clupea harengus* L.) were ripened for 1–21 days (4 samples) in brine (14% NaCl, 0.7% acetic acid), 5 kg of fish in each, sample to brine ratio 1:1 (*w*:*w*) and at a temperature of 8 ± 1 °C [15]. They were then skinned, and meat was disintegrated with a household mincer.

### 2.2. TCA Extracts from Meat and Brine

The extracts from fresh and salted meat herring were prepared by homogenization of 10 g of minced meat with 50 mL of 6.5% trichloroacetic acid (TCA) for 1 min at 24,000 rpm (mechanical homogenizer H500, Pol-Eko-Aparatura, Wodzisław Śląski, Poland). Homogenate was left for 30 min and again homogenized. After 30 min, the homogenate was centrifuged at 10,000× *g* for 10 min. In turn, 10% TCA was added to the brine (1:1, *v*:*v*), mixture was left for 30 min and filtered. In meat and brine extracts final TCA concentration was 5%. Prior to CZE separation, the TCA extracts of meat and brine were diluted 3 times with ultra-pure water and filtered by poly-ethersulfone syringe filter [29].

### 2.3. Capillary Electrophoresis

The TCA extracts were separated with capillary zone electrophoresis (BioFocus Capillary Electrophoresis System 2000, Bio-Rad, Hercules, CA, USA) with spectrophotometric detection (DAD detector, UV range) at 200 nm directly on the capillary [28]. Uncoated fused-silica capillaries (Bio-Rad, 148-3060), 32 cm effective length (to the detection window) and 50 μm inner diameter, were used. Prior to each assay and among runs, a capillary was rinsed for 5 min with the running A 0.1 M phosphate buffer, pH 2.5 (Sigma-Aldrich, Darmstadt, Germany, P-2188). At the end of each assay day, capillary was rinsed with washing buffer (containing NaOH) for 1 min (BioRad, 148-5022), then with ultra-pure water (1 min) and buffer A (5 min). The assays were run using pressure injections 5 psi·s. Separation was carried out at a constant voltage of 20 kV, polarity + to −, and constant capillary temperature of 35 °C, with the running buffer A. The separation of a single sample took 25 min. The data were collected and processed by the BioFocus 2000 system integrator.

### 2.4. Analyses of Meat Moisture, Salt and Total Nitrogen Content

Moisture, salt and total nitrogen contents in meat and brine were determined using standard AOAC analytical techniques no. 950.46B, 976.09, and 940.25, respectively.

### 2.5. Non-Protein Nitrogen (NPN) Fractions Analysis

In 5% TCA extracts from meat and brine [28] were determined: (i) non-protein nitrogen with the Kjeldahl’s method (AOAC no. 940.25); (ii) protein hydrolysis products: peptides (HP(R)) and amino acid (PHP(A)), with the modified Lowry method [10].

### 2.6. Sensory Assessment of Salted Meat Texture

The salted herring fillets were analyzed by sensory profiling performed by 7 person sensory panel trained in the analysis of salted herrings according to ISO 11035 [31] using a five-point unstructured scale with 0.5 points accuracy anchored at their extremes with minimum and maximum degrees of acceptance [32]. A higher note signifies better texture attributes (1 point the worst texture/disliked extremely, 5 points the best texture/liked extremely). Briefly, three skinned fillets from each sample were served in porcelain trays. Each assessor was cut three pieces (app. 3 cm width each), one from each fillet, to test them. Tested area of fillets was in a range from 2/10 to 6/10 fillets length measured from head side. The evaluations were performed in separate boxes under daylight and at ambient temperature. The assessors used water and flat bread to clean their palate between samples. The texture attributes and their definitions according to ISO 5492 [33] are shown in Table 1.

### 2.7. Hardness Parameter of Meat

The 4 fillets from each sample were analysed with a TA-XT 2/25^®^ Texture Analyzer (Stable Micro Systems, Godalming, UK). The test included two-fold penetration of a cylindrical pin P10 (10 mm diameter), with sample deformation up to 50% of height at the speed of 5 mm·s^−1^. The course of the test was recorded as curves representing changes of force in time. The tests were conducted for each fillet separately (in 3 repetitions each). Tested area of fillets was in a range from 2/10 to 6/10 fillets length measured from head side.

### 2.8. Statistical Analysis

All analyses were performed in three replications. Results were analyzed statistically using one-way analysis of variance (ANOVA), Principal Component Analysis (PCA) and cluster analysis with Statistica 13.0 (StatSoft, Tulsa, OK, USA). The PCA was used to investigate correlations between sensory analyses and all proteolysis indicators [34,35]. The multi-way cluster analysis was employed to group the capillary electrophoresis indicators depending on salting time of herring fillets [36,37]. The ANOVA *p* value was set at 0.05, and the differences between treatments were examined using the post hoc test Tukey’s honestly significant differences (*p* < 0.05) [38]. In MS Excel, 3 types of regression (linear, logarithmic and power) were each determined for area-peak and height-peak of amino acids, but the correlation with the highest R2adj value was given. The model fit and the performance were evaluated using the adjusted determination coefficient (R2adj), and the root mean square error (RMSE), respectively.

## 3. Results and Discussion

### 3.1. Protein Hydrolysis Products

**Figure 1:**
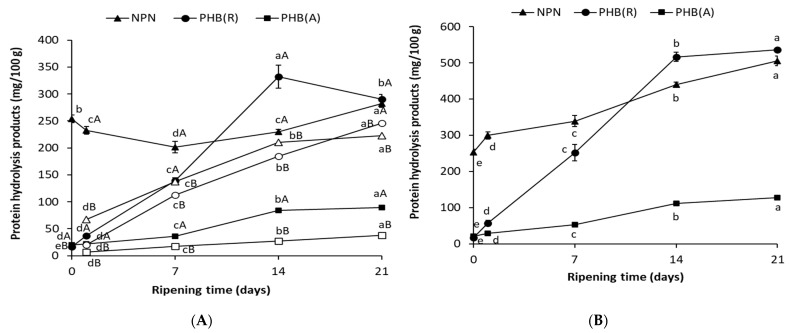
Content of non-protein nitrogen (NPN), peptides fraction (PHP(R)) and amino acid fraction (PHP(A)) in (**A**) meat (black shape), brine (white shape) and (**B**) sum of meat and brine during salting of herring. ^a–e^ Ripening times with the same lowercase letter separately for each nitrogen fraction differ insignificantly (*p* < 0.05); ^AB^ No significant differences were found in meat and brine with the same letter (*p* < 0.05).

During salting various changes occur that alter the chemical and physicochemical properties of fish. In general, the changes can be divided into two phases, salting and ripening. The first stage, in which salt diffuses from the brine into the meat and the salt content of the meat stabilizes, takes 1–2 days [39]. In the second step, proteolysis of proteins occurs under the influence of proteases whose activity changes according to the pH value, salt concentration and temperature. Therefore, the process of ripening of salted herring is often described by changes in soluble non-protein nitrogen (NPN) compounds and selected protein hydrolysis products (PHP). Results show, that during the first week of salting, the non-protein nitrogen content of meat decreased from 254 mg to 233 mg/100 g (Figure 1A), probably due to diffusion of amino acids and peptides to the brine [1,20]. During subsequent weeks of salting, the NPN content increased, reaching 282 mg/100 g. There were 67 mg of NPN in 100 mL of brine after only 1 day of salting. NPN content increased until day 14 of salting (211 mg), after which the increase was not statistically significant (Figure 1B). The content of peptide fraction in meat also increased intensively but only until day 14, reaching 332 mg/100 g, while after 3 weeks the content decreased to 290 mg. On the other hand, the content of the amino acid fraction in meat increased until the 21st day of salting, reaching 90 mg (Figure 1A). In brine, the fraction of peptides and amino acids during salting increased according to a linear function reaching, after 3 weeks, 246 and 38 mg, respectively. This may indicate that intense endopeptidase activity and substrate formation for exopeptidases continued until week 2. Szymczak [19] showed that the marinating stage of herring meat started with a dominance of endopeptidase activity, mainly cathepsin D, whose activity increased rapidly up to 2–4 days and was manifested by peptide formation. The activity of exopeptidases in the marinades was initially marginal but increased continuously and a high content of amino acid fraction was formed after one week of ripening. The results in Figure 1 show that for the present method of salting of herring fillets with acetic acid, the increase in endopeptidase activity is prolonged compared to marinades [19,22,23]. The reason was the high concentration of salt (cathepsin D inhibitor), whose presence was not balanced by a sufficiently high concentration of acetic acid (cathepsin D catalyst) [19]. As a result, the rate of change in the quantitative and qualitative composition of amino acids may be different compared to the traditional salting or marinating method.

**Figure 2:**
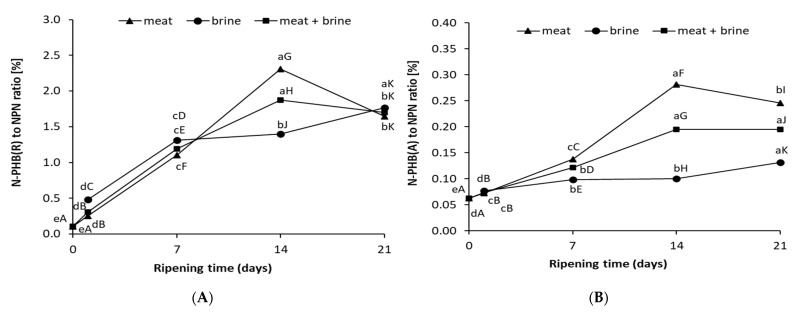
(**A**) Nitrogen of PHP(R) to NPN ratio and (**B**) PHP(A) to NPN ratio determined in meat and brine during salting of herring. ^a–e^ Ripening times with the same lowercase letter separately for each nitrogen fraction differ insignificantly (*p* < 0.05); ^A–K^ No significant differences for each nitrogen fraction were found in meat and brine with the same letter (*p* < 0.05).

Szymczak et al. [1] showed that a useful indicator of the marinade ripening and nitrogenous compound losses is the ratio of amino acid and peptide fraction nitrogen to non-protein nitrogen. In this study during herring salting, the ratio of N-PHP(R) in the NPN meat increased to 2.3% by day 14 and then decreased to 1.7% (Figure 2A). In brine, the ratio of N-PHP(R) increased for 3 weeks of salting, but after week 2 the growth rate was lower than in meat. In contrast, the ratio of N-PHP(A) was 10 times lower than that of N-PHP(R) and the differences between meat and brine were significant after one week of salting (Figure 2B). The ratio of N-PHP(A) in meat increased until day 14 reaching 0.28% and decreased after week 3 of salting. In brine, the ratio of N-PHP(A) increased until week 3 of salting, reaching 0.13%. The results showed that PHP nitrogen to NPN ratio changed during salting, very similar to the PHP fraction content. Correlation analysis showed an almost full positive correlation (0.970) between PHP indicators and medium correlation of these indicators relative to NPN (0.442–0.644).

### 3.2. CZE Amino Acid Separations

**Figure 3:**
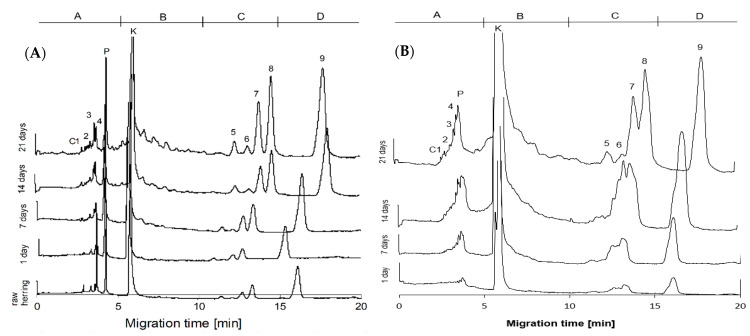
Electrophoregrams of TCA extract of (**A**) raw herring meat and after 1–21 days of salting and (**B**) brine. C1—creatinine, 2—lysine, 3—arginine, 4—histidine, P—peptide GGYR, K—creatine, 5—tryptophan, 6—methionine, 7—phenylalanine, 8—tyrosine, 9—cysteine/cystine.

CZE separation of the TCA extracts from raw and salted herring meat (Figure 3A) and brine (Figure 3B) gave electro-phoregrams with increasing height of peaks during time of ripening. The electro-phoregrams were divided into four fields depending on migration time: A (0–5 min), B (5–10 min), C (10–15 min), and D (15–25 min), according to Felisiak [28]. The quantity and size of peaks in areas A and B increased intensively with ripening time, indicating an increase in dipeptides and free neutral amino acids. The greatest changes during herring salting concerned height and area of peaks in C–D electro-phoregram fields. In area C, after 21 days of ripening, peak heights increased 6–10 times in meat and 1.5–13 times in brine. There are four main peaks which were identified as Trp, Met, Phe, Tyr, and also some smaller peaks of Gln, Glu, Thr [29]. Creatine and creatinine have very large peaks on the electro-phoregrams, making them very easy to determine using CZE. Despite this, these compounds were not included for the determination of ripening indicators, because very often unique results of the quantitative content of creatine and creatinine were obtained. The reason was conversion of creatine into creatinine catalyzed by acids in extract [40]. The amino acids that are formed in the highest concentration during herring salting and have sufficiently high absorbance were then selected from the electrophoregrams. Using CZE without derivatisation, the determination of basic and hydrophobic amino acids was not difficult, but the determination of acidic amino acids was difficult due to the low UV absorbance of these substances. Therefore, too large values of the standard deviation of the results and too large statistical uncertainty of the Kjesvaar indicator were obtained by the CZE method. Nevertheless, the calculations made showed that, during ripening, the Kiesvaar indicator for meat (basic to acidic amino acids) decreased from 4 to 0.5 after reaching consumptive ripeness (data not shown). Finally, to determine the ripeness indicators for salted herring, for the meat samples, the essential amino acids were selected: lysine, arginine, histidine, and the hydrophobic tryptophan, methionine, phenylalanine and tyrosine. For brine samples, CZE separation was less selective than for meat (Figure 3) and method optimisations were used [28], allowing accurate measurements of the height and area of selected peaks [29].

Plorin and Lenowa [41] in a well ripened herring determined that several amino acids were released, such as aspartic and glutamic acids, alanine, and phenylalanine. Kiesvara [3] showed that the ratio of basic amino acids (Lys, His, Arg) to the acidic amino acids (Asp, Thr, Ser, Glu) and proline decreased when herring was ripened with digestive enzymes alone. This indicator was not confirmed by Stefansson and Stefansson [42] in heading herring and fillets during salting. Later results from Gringer et al. [6] showed that the quantitative and qualitative composition of free amino acids in fish salted using the traditional method differed from those salted with vinegar. The traditional brine after salting contained the most lysine and threonine, while the second method contained valine and leucine. Of the non-essential amino acids, the brine with vinegar contained the most aspartic and glutamic acid and serine. High concentrations of these amino acids have been confirmed by studies of Beaulieu et al. [43]. Özden [18] also found that ripening of herring (anchovy) fish meat using endogenous muscle proteases promotes the formation especially of aspartic acid, threonine, proline, glycine, tyrosine, lysine, and serine. The formation of large amounts of these amino acids is important for consumers because histidine, tryptophan, tyrosine, proline, glycine, alanine, cysteine, lysine, and methionine have particularly high antioxidant activity [44,45].

The height and area of the selected peaks and their interrelationships in meat or brine, respectively, were analysed (Table 2 and Table 3). Sixteen indicators were developed based on the CZE electro-phoregrams. The height and area of the hydrophobic (HAA) and basic (BAA) amino acid peaks in both meat and brine increased during ripening (Figure 3). The peak area and peak height for HAA and BAA increased in meat according to a linear function. The increase in HAA-area was 20 times greater than that of the BAA-area, while the increase in HAA-height was 9 times greater than BAA-height (Table 2). The area or height of BAA to HAA ratio decreased during salting according to a logarithmic function, while HAA to BAA ratio increased according to a power function. In the case of brine, the area and peak height for HAA also increased linearly, while for BAA they increased powerfully (Table 3). The regressions for HAA to BAA or BAA to HAA area ratio during salting were linear functions, but the regression fitting (R2adj) was average. The regressions of alkaline and hydrophobic peak heights ratio had a higher fit value. The results showed that the area and height changes for hydrophobic amino acids had a higher angular coefficient (slope) value, and the regression was stronger than that for basic amino acids. Therefore, the relationship between Phe and Tyr, which had the most separated peaks on the electro-phoregrams, was checked. Logarithmic and linear regressions for the area and peak height of Phe to Tyr ratio were very high in meat (Table 2) and weak and medium in brine (Table 3). Of the basic amino acids, the greatest selectivity of the CZE method was for histidine and arginine. These amino acids were compared with tyrosine. Negative regressions of area and height of His to Arg ratio were logarithmic in meat and linear in brine. Similar regressions in meat and brine were obtained for the proportion of area and peak height of His + Arg to Tyr (Table 2 and Table 3). The results showed that, using the CZE method, the ripeness indicator of salted herring can be determined as the ratio of the area or height of the peaks corresponding to basic hydrophobic amino acids, or the inverse relationships.

In order to compare the models fit and the performance, R2adj and RMSE values were calculated. R2adj values varied from 0.298 to 0.998 for meat and 0.357–0.991 for brine, indicating that not all models were suitable for describing ripening behavior in herring fillets during salting. Analyzing the RMSE, the models presented a good performance in all indicators of the salting ripening: 0.009 ≤ RMSE ≤ 0.475 for meat and 0.015 ≤ RMSE ≤ 0.482 for brine. The exception was the large RMSE values for the BAA and HAA total area and height indicators but, as a percentage of the source results, these were also low (Table 2 and Table 3). From the indicators presented, the strongest fit (R2adj) and simultaneously the lowest model errors (RMSE) for meat were: Tyr to Phe height ratio, HAA to BAA height ratio, Tyr to Phe area ratio, BAA to HAA area ratio; whereas for brine: His to Tyr height ratio, His + Arg to Tyr height ratio, Phe to Tyr area ratio, BAA to HAA height ratio. The best regressions for meat were not repeated for brine. This is likely to be due to the different susceptibility of amino acids to (i) diffusion from meat into brine and (ii) extraction of amino acids into TCA. In meat, the best ripening indicators usually had logarithmic regressions, while in brine more regressions were linear. The selected indicators are mostly concerned with the sum of hydrophobic and basic amino acids or consider only the relationships for tyrosine, phenylalanine, histidine, and arginine.

### 3.3. Sensory Evaluation and Meat Hardness vs. Ripening Indices

**Figure 4:**
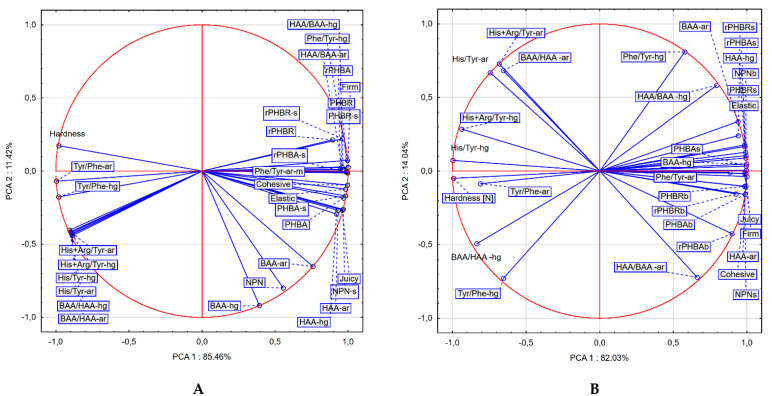
PCA biplot for correlation between sensory assessment and hardness vs proteolysis indicators determined in (**A**) meat and (**B**) brine. -ar—area of peak; -hg—height of peak; -s—sum of fraction content in meat and brine; r-—ratio of PHP(A) or PHP(R) nitrogen to NPN.

The results of sensory evaluation showed that salted herring meat reached industrial ripeness (semi-finished product) after 14 days, while after 21 days the meat had consumer ripeness (full ripeness). The greatest changes in meat texture during salting were found for the firm, cohesive and elastic parameters, while the least effect of salting was found for the juicy parameter (Table 4). Meat texture during industrial ripeness of salted herring was rated between 4.25 and 4.50 points, while during consumer ripeness it was rated above 4.62 points. From 1 to 14 days of salting, the texture evaluation of herring meat increased faster than after 14 days. Between days 14 and 21, the differences were statistically significant only for the firm parameter. As the sensory evaluation of texture increased, the value of the meat TPA-hardness parameter decreased from 5.5 to 4.1 N (Table 4). The correlations between sensory evaluation parameters were checked by PCA analysis (Figure 3). Despite the different rates of change in sensory evaluation (Table 4), the parameters correlated very strongly with each other (Figure 4A). Also, TPA-hardness had an almost full negative correlation with sensory parameters: elastic (−0.998), juicy (−0.995), cohesive (−0.990), and firm (−0.970). Lisiecki [46] showed that sensory evaluation of softness during ripening of salted herring meat had a very strong correlation with texture profile parameters, especially TPA-hardness.

**Figure 5:**
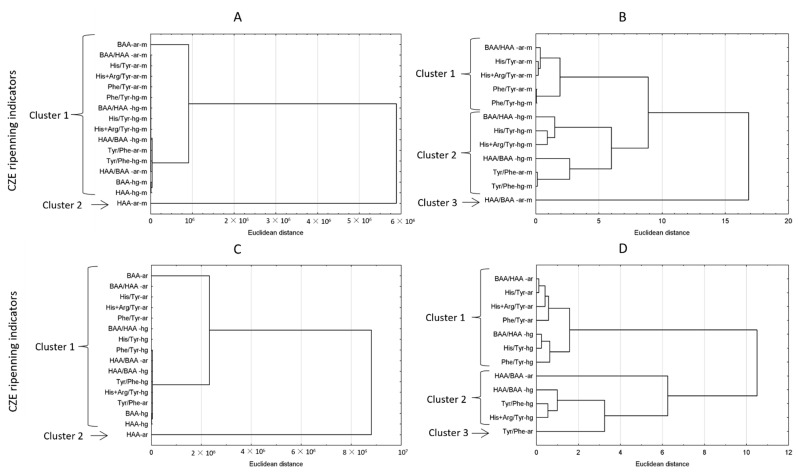
The grouping of CZE ripening indicators depending on occurrence in (**A**,**B**) meat and (**C**,**D**) brine during herring salting. -ar—area of peak; -hg—height of peak; -m—results for meat.

The analysis of the correlation of ripening indicators with sensory evaluation parameters and TPA-hardness of salted fillets started with PCA (Figure 4). The obtained correlations were explained by axis 1 and 2 in 97% for meat and in 96% for brine. In the PCA plot, the ripening indicators determined in meat (Figure 4A) were more closely related to each other than those determined in brine (Figure 4B). For better characterization, an additional cluster analysis was performed (Figure 5). Cluster analysis grouped the CZE indicators into two main groups and two additional groups below the cutoff line that are not visible due to the scale of the axis (Figure 5A,C). In meat and brine, the HAA-area indicator belonged to the first cluster, and the other indicators were included in the second cluster (Figure 5A,C). The large Euclidean distance indicates a lack of similarity between HAA-ar and the other CZE indicators, more so in meat than in brine. In order not to interfere with the grouping of indicators with high values, the analysis was repeated without their participation and the indicators were divided into three clusters (Figure 5B,D). Meat in the first cluster was dominated by peak area indices, the second cluster was dominated by peak height indices, and the third cluster was HAA/BAA-area ratio (Figure 5B). The indicators in cluster 1 obtained from peak area had three times closer distance to each other than the indicators obtained from peak height—cluster 2. In contrast, the third cluster (HAA/BAA ratio) was strongly distant to clusters 1 and 2. For brine, the HAA/BAA-area indicator also formed a separate cluster, while the other two clusters contained both indicators based on area and peak height (Figure 5D). The best ripening indicator Phe/Tyr-hg in meat according to the cluster analysis was the least distant from His/Tyr-ar and His + Arg/Tyr-ar indicators. In contrast, the best His/Tyr-hg ripening indicator in brine was the least distant from the BAA/HAA-hg and Phe/Tyr-hg indicators. This shows that these indicators, especially Phe/Tyr occurring simultaneously in meat and brine, can provide similar knowledge of herring ripening. On the other hand the results showed that the same indices for meat and brine were grouped differently despite high similarities. The classical NPN and PHP indicators in meat and brine, despite similar changes in content over time, also have different usefulness for assessing meat ripening. This is probably why the PCA plots for meat and brine obtained different alignments/correlations of the tested indicators.

The results showed that industrial ripeness of salted fillets occurred on the day of slowing down of the dynamics or stopping of the growth of PHP(R) content in meat and NPN content in brine (Figure 2). This phenomenon also occurred for N-PHP(R) or N-PHP(A) in NPN ratio calculated in meat or in meat + salt (Figure 3). Meat NPN strongly correlated with juicy (0.731) parameter and TPA-hardness (−0.657). Perez-Villarreal and Pozo [47] proposed the indicator as non-protein nitrogen, the level of which increased during the ripening of anchovies. Despite this, the indicator was not popular due to diffusion of nitrogen into the surrounding brine [48]. Our results showed that the NPN content in brine or the sum of NPN meat + salt was a better indicator because it had almost full correlation with sensory evaluation parameters and TPA-hardness (0.955–0.996). Peptide and amino acid fractions and their contribution to NPN in meat and brine correlated as strongly with sensory evaluation of salted herring (Figure 4A,B). A relation between the texture of the herring and the biuret value was found in the brine [49]. These indicators have not been adopted in industry because they are very labor and cost intensive and require a large sample mass for analysis.

Among the proposed indicators of ripeness degree of salted herring based on free amino acids determined by the CZE method, the strongest correlation for firmness in sensory assessment and for hardness in TPA was Phe/Tyr-height in meat 0.998 and −0.987, respectively, while in brine His/Tyr-height (−0.990) and HAA-area (−0.999). Regression model analysis showed that the Phe/Tyr-height ratio in meat had a R2adj = 0.983 and an RMSE error of 0.309. In contrast, the His/Tyr-height indicator in brine had better values: a larger R2adj = 0.991 and a many times smaller error value RMSE = 0.015 (Table 3 and Table 4). The value of Phe/Tyr-height-meat ratio increased during salting of herring according to a logarithmic function. When herring meat reached industrial ripeness the value of this indicator was 64% (0.64), while for salted herring with consumer ripeness it was 68.5% (Table 3). For the His/Tyr-height-brine indicator, its value decreased when the herring was salted according to a linear function. For industrial ripeness, the brine indicator value was 62.7% and for consumer ripeness it was 52.1%. The results showed that the indicator in brine, compared to the indicator in meat, had a larger difference between industrial and consumer ripeness in addition to better regression parameters. This was due to the difference in the rate of change of individual amino acid content during salting of herring. For these reasons, the His/Tyr-height-ratio in brine most accurately indicated the changes in ripening of salted herring fillets with acetic acid addition method.

## 4. Conclusions

Salted fish are still popular, so the industry is optimizing production technology for the available raw material. These changes affect the meat ripening process and the quantitative and qualitative composition of protein hydrolysis products (PHP), which are indicators of the dynamics of the salting process. CZE analyses in meat and brine were performed in less than 1 h, which is satisfactory for industry. A cheap and rapid CZE method without derivatization allowed the determination of hydrophobic and basic amino acid groups. Sixteen indicators were developed based on the height and peak area of the amino acids. Traditional NPN indicators were also examined in meat and brine, which correlated strongly with the results of sensory analysis of meat texture and TPA-hardness. Statistical regression analysis (strength of fit and magnitude of error) and correlation analysis by PCA showed that CZE indicators were significantly more precise than traditional NPN indicators. The CZE indicators obtained for brine extract were significantly better than the CZE indicators obtained from the meat results. Performing CZE analysis of brine was also easier than meat. Most of the indicators for brine were in the form of linear regressions, which are easier to understand than the logarithmic and power regressions more commonly obtained in meat.

The results showed that capillary electrophoresis can be successfully used to follow proteolytic changes during the ripening of salted herring by a new method with the addition of acetic acid. It is possible to separate TCA extracts without additional processes, only after dilution of the sample. CZE is very fast, and the use of equally fast extraction allows changes in the ripening herring to be tracked in real time in industry, without the great delay that results from the time-consuming analyses by other methods.

## Figures and Tables

**Table 1:** Sensory attributes of texture assessed in the present study.

Texture Attribute	Description
Cohesive	Force requited to spread the meat in the mouth
Juicy	Amount of juice released during mastication
Firm	Force required to compress the sample between the molar teeth
Elastic	The ability of the sample to regain its form after the first compression by the molars

**Table 2:** Ripening indicators of salted herring based on free amino acids in meat. BAA—sum of basic amino acids; HAA—sum of hydrophobic amino acids; r-—amino acid to amino acid ratio; -ar—area of amino acid peak; -hg—height of amino acid peak.

Indicator of Ripening	Time of Ripening [day]				Regression [y= ]	R2adj	RMSE
	1	7	14	21			
BAA-ar	213,610	178,114	256,026	313,165	5746x + 178460	0.732	26,056
HAA-ar	304,327	875,943	1,488,453	2,680,218	115,941x + 90,870	0.973	145,774
r-BAA/HAA-ar	0.702	0.203	0.172	0.117	−0.196ln(x) + 0.673	0.952	0.052
r-HAA/BAA-ar	1.42	4.92	5.81	8.56	1.4581x^0.5698^	0.985	0.475
BAA-hg	4903	3288	4654	5856	66.89x + 3957	0.298	769
HAA-hg	1772	4496	7637	14123	602.6x + 528	0.963	883
r-BAA/HAA-hg	2.767	0.731	0.609	0.415	−0.792ln(x) + 2.642	0.948	0.218
r-HAA/BAA-hg	0.36	1.37	1.64	2.41	0.3719x^0.6066^	0.986	0.132
r-Phe/Tyr-ar	0.467	0.544	0.639	0.656	0.0098x + 0.471	0.931	0.020
r-Phe/Tyr-hg	0.467	0.580	0.640	0.685	0.0691ln(x) + 0.461	0.983	0.309
r-Tyr/Phe-ar	2.140	1.839	1.566	1.525	−0.209ln(x) + 2.163	0.961	0.049
r-Tyr/Phe-hg	2.140	1.723	1.563	1.460	−0.224ln(x) + 2.145	0.998	0.009
r-His/Tyr-ar	0.964	0.185	0.157	0.087	−0.296ln(x) + 0.913	0.937	0.089
r-His/Tyr-hg	3.788	0.665	0.543	0.317	−1.178ln(x) + 3.575	0.934	0.368
r-His + Arg/Tyr-ar	1.05	0.29	0.26	0.18	−0.291ln(x) + 0.999	0.940	0.084
r-His + Arg/Tyr-hg	4.33	1.10	1.00	0.68	–1.225ln(x) + 4.116	0.938	0.369

**Table 3:** Ripening indicators of salted herring based on free amino acids in brine. BAA—basic amino acid; HAA—hydrophobic amino acid; r-—amino acid to amino acid ratio; -ar—area of amino acid peak; -hg—height of amino acid peak.

Indicator of Ripening	Time of Ripening [days]				Regression [y= ]	R2adj	RMSE
	1	7	14	21			
BAA-ar	105,934	303,130	930,567	772,641	100,357x^0.7114^	0.927	154,493
HAA-ar	318,523	1,155,109	2,992,610	3,638,395	176,117x + 132,903	0.967	243,400
r-BAA/HAA-ar	0.333	0.262	0.311	0.212	−0.0047x + 0.329	0.536	0.031
r-HAA/BAA-ar	3.01	3.81	3.22	4.71	0.0672x + 2.963	0.583	0.425
BAA-hg	1135	3241	7106	8096	1080x^0.6614^	0.977	570
HAA-hg	1454	4795	12619	12573	612.8x + 1273	0.886	1650
r-BAA/HAA-hg	0.781	0.676	0.563	0.644	−0.058ln(x) + 0.777	0.764	0.038
r-HAA/BAA-hg	1.28	1.48	1.78	1.55	1.2864x^0.0847^	0.720	0.105
r-Phe/Tyr-ar	0.364	0.296	0.565	0.645	0.0176x + 0.282	0.784	0.066
r-Phe/Tyr-hg	0.607	0.721	1.039	0.716	0.0834ln(x) + 0.613	0.357	0.131
r-Tyr/Phe-ar	2.750	3.380	1.768	1.550	3.349e^−0.038x^	0.721	0.482
r-Tyr/Phe-hg	1.647	1.387	0.962	1.397	−0.149ln(x) + 1.631	0.490	0.178
r-His/Tyr-ar	0.393	0.345	0.366	0.212	−0.0079x + 0.414	0.715	0.037
r-His/Tyr-hg	0.933	0.795	0.627	0.521	−0.0209x + 0.944	0.991	0.015
r-His + Arg/Tyr-ar	0.553	0.476	0.533	0.304	−0.0103x + 0.578	0.627	0.060
r-His + Arg/Tyr-hg	1.51	1.44	1.23	0.95	−0.0281x + 1.585	0.951	0.046

**Table 4:** Sensory assessment of texture (points) and TPA-hardness (N) of herring meat during salting.

Analyses	Parameter	Salting Time [day]			
		1	7	14	21
Sensory assessment	Cohesive	2.85 ± 0.1 ^c^	3.5 ± 0.2 ^b^	4.3 ± 0.15 ^a^	4.7 ± 0.21 ^a^
	Juicy	4.0 ± 0.2 ^b^	4.1 ± 0.25 ^ab^	4.5 ± 0.1 ^a^	4.65 ± 0.1 ^a^
	Firm	1.8 ± 0.2 ^d^	3.2 ± 0.1 ^c^	4.25 ± 0.1 ^b^	4.8 ± 0.1 ^a^
	Elastic	3.2 ± 0.0 ^c^	3.6 ± 0.1 ^b^	4.5 ± 0.0 ^a^	4.62 ± 0.15 ^a^
TPA	Hardness	5.72 ± 0.71 ^a^	5.33 ± 0.45 ^a^	4.35 ± 0.40 ^b^	4.11 ± 0.38 ^b^

^a–d^ Means within same row with the same common lowercase latter differ insignificantly (*p* < 0.05).

## Data Availability

The datasets generated for this study are available on request to the corresponding author.
